# Association Between Fulfilment of Expectations and Health-related Quality of Life after Gastric Bypass

**DOI:** 10.1007/s11482-012-9175-9

**Published:** 2012-08-24

**Authors:** S. G. Pristed, H. K. Omar, J. P. Kroustrup

**Affiliations:** 1 Department of Endocrinology, Aalborg Hospital – Aarhus University Hospital, Moelleparkvej 4, Medicinerhuset, 9100 Aalborg, Denmark; 2 Department of Surgery, Aalborg Hospital – Aarhus University Hospital, Hobrovej 18-22, 9100 Aalborg, Denmark

**Keywords:** BMI, Expectations, Gastric bypass, Health-related quality of life, SF-36

## Abstract

The objective was to examine the relationship between fulfilment of expectations and health-related quality of life 4 and 12 months after gastric bypass. A follow-up study based on patients undergoing gastric bypass at Aalborg Hospital – Aarhus University Hospital during February 2008 to December 2009. Health-related quality of life was assessed by Short Form 36 and summarized into the physical component summary and the mental component summary. Information on expectations was questionnaire based. Associations were analysed by linear regression. Included were 87 gastric bypass patients. Compared with patients with fulfilled expectations having expectations partly fulfilled −7.3 (−11.3; −3.3) or not having expectations fulfilled −11.2 (−18.8 ; −3.5) was associated with low a mental component summary 4 months after surgery. At 12 months follow-up patients who reported not to have expectations fulfilled had a low mental component summary score −16.3 (−26.5; −6.2) when compared to their counterparts with fulfilment of expectations. Not having expectations to changes in general well-being fulfilled is associated with low mental component summary. This is seen at follow-up points 4 and 12 months after gastric bypass.

## Introduction

The prevalence of obesity is alarming in many parts of the world (IASO 2009; World Health Organization 2000). Besides being associated with morbidity (Guh et al. 2009) obesity is found to be associated with low health-related quality of life compared to national norm data (Choban et al. 1999; Dixon et al. 2001; Larsson et al. 2002; Pristed et al. 2011; Victorzon et al. 2010). Bariatric surgery has become the choice of reducing excess weight for more and more people and bariatric surgery results in greater weight loss than conventional weight loss programs for individuals classified as being obese (Colquitt et al. 2009). In the Swedish Obesity Study recovery from diabetes, hypertriglyceridaemia, low levels of high-density lipoprotein cholesterol, hypertension and hyperuricaemia were more favourable in the surgery group than in the control group. Furthermore, the 2- and 10-year incidence rates of diabetes, hypertriglyceridaemia and hyperuricaemia were lower in the surgery group than in the control group (Sjøstrom et al. 2004). In addition patients who undergo gastric bypass surgery have decreased long-term all-cause mortality and from disease specific causes as compared with control subjects with a self-reported body mass index of at least 35 kg/m^2^ (Adams et al. 2007). Besides controlling current medical problems obese individuals seek bariatric surgery to improve their health-related quality of life (Choban et al. 1999; Dixon et al. 2001; Larsson et al. 2002).

**Fig. 1:**
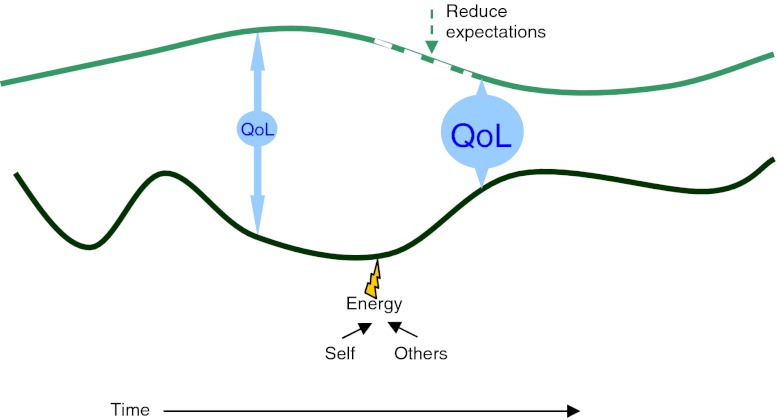
The Expectations model (Calman 1984)

The expression quality of life is intuitively understood by most people, and yet no exhaustive definition exists. To narrow the focus into effects of health, illness and treatment the term health-related quality of life is broadly accepted. Health-related quality of life is a multifaceted construct assessed by questionnaires (Testa and Simonson 1996) and approached from various perspectives (Sirgy 2001). Generally the questionnaires are divided in two sections; disease specific or generic (Patrick and Deyo 1989). Conceptual models of quality of life suggest the construct to reflect differences between an ideal state and the current state of the individual (Calman 1984; Sirgy 1986; Testa and Simonson 1996). In the expectations model, described by Calman et al. a gap between banks with reality on one side and hopes and expectations on the opposite side has been used as a metaphor for quality of life, Fig. 1. According to the model, quality of life is improved by modifying expectations or by putting energy into changing the current position. In agreement with this Testa et al. explain that two individuals with the same health status may report different health-related quality of life because of differences in their expectations and ability to cope with limitations (Testa and Simonson 1996).

The differences between an ideal state and the current state become evident when considering bariatric patients’ weight loss expectations as a result of bariatric surgery. High or unrealistic expectations to weight loss have previously been described among gastric bypass patients (Karmali et al. 2011). Instead of aiming at what is obtainable as a result of gastric bypass, the dream weight corresponds to current weight classifications, which implies a weight loss approximating 100 % of the overweight (Foster et al. 2001; Karmali et al. 2011; Wee et al. 2006; White et al. 2007). As far as we know no previous studies have described the association between fulfilled expectations and health-related quality of life among gastric bypass patients.

Understanding the potential contributors to low health-related quality of life may be a central part in settings trying to improve health-related quality of life. We hope to add knowledge to the applied research in health-related quality of life by testing the hypothesis:

Fulfilment of expectations to changes in general well-being as a result of weight loss is associated with health-related quality of life.

## Methods

### Patients

The study was conducted as a prospective cohort study of morbidly obese subjects with follow-up 4 and 12 month after gastric bypass. The baseline questionnaires were filled in at the Department of Medical Endocrinology before the medical examination. At follow-up the questionnaires were sent by mail to the patients together with an explanatory letter and a stamped addressed envelope. In total 238 patients who had a medical examination in the period between January 2008 and September 2009 at the Department of Medical Endocrinology, aiming at evaluating the suitability for bariatric surgery were invited to participate. The criteria for surgery were age between 20 and 60 years, BMI ≥ 40 kg/m2 or ≥ 35 kg/m2 with obesity-related morbidities, and no serious illness, former alcohol or drug abuse or active psychosis. Patients who did not have gastric bypass at the public university hospital were excluded from the study. At 4 months follow-up 136 patients were eligible for follow-up and at 12 months follow-up 105 patients were eligible for follow-up. After excluding patients with incomplete data a total of 87 patients were included in the analysis.

### Operative Procedure

All operative procedures were performed by a team of experienced surgeons working with the same medical team of endocrinologists, gastroenterologists, dieticians and nurses. Gastric bypass is a combined method of restriction and malabsorption. A small upper gastric pouch is created. The small intestine is divided at the midjejenum and the distal end is anastomosed to the gastric pouch and referred to as the Roux limb. The distal portion of the stomach and the biliopancreatic limb are anastomosed down the jejunum, thus pancreatic and biliary secretions are in contact with food below this anastomosis. The greater the bypassed small intestine the less nutrient uptake will occur (Demaria 2007).

Some early complications of gastric bypass include gastrointestinal bleeding, infection, thromboembolism and anastomotic leaks and the late complications include internal hernia, marginal ulceration, anastomotic stricture and malabsortion (Al Harakeh 2011).

### The Questionnaires

#### Short Form 36

The generic questionnaire Short Form 36 (SF-36) was used to assess health-related quality of life at baseline and at follow-up points of 4 and 12 month. The questionnaire has been described in details elsewhere (Bjorner et al. 1997). Health-related quality of life is summarized into the physical component summary and the mental component summary after standard procedure (Bjorner et al. 1997), Fig. 2.

**Fig. 2:**
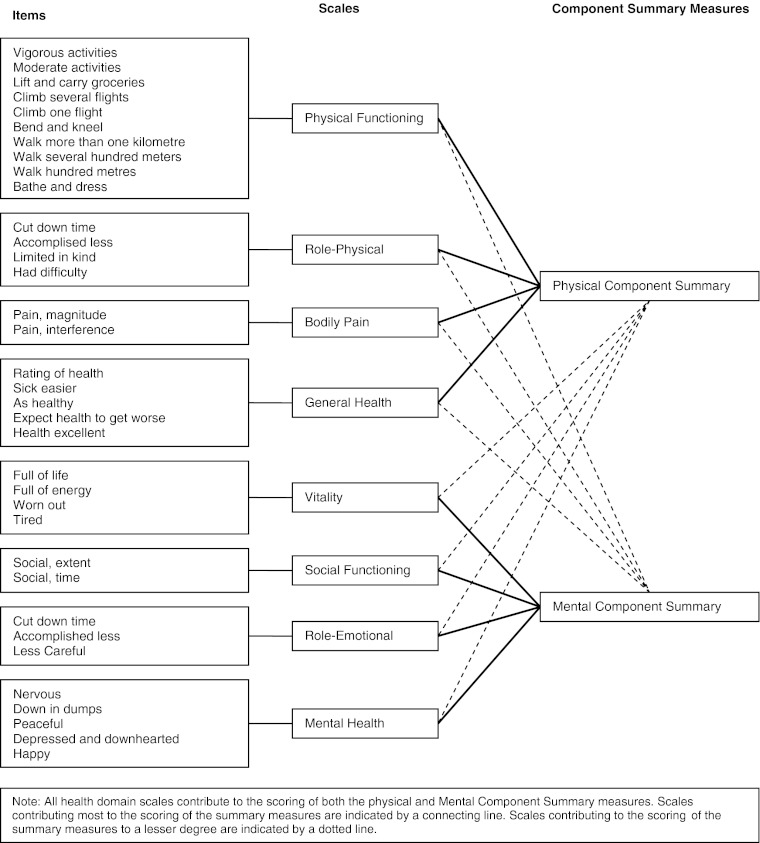
Short Form -36

#### Obesity Specific Questions

The obesity specific questions concern six aspects of general well-being: State of health, physical activity, emotional problems, pain, social or familial problems and work capacity. For each of the aspects the subjects answered questions about their attribution of impairments to morbid obesity and expectations to changes as a result of weight loss. At follow-up the subjects were asked about the degree of fulfilment of expectations. In this study the term general well-being will be used when referring to the obesity specific questions. The questions are illustrated in Table 1.

**Table 1:** Obesity specific questions at baseline and follow-up

Baseline	
Aspects of general wellbeing	Attributions of impairments to morbid obesity
I) State of health	a) The overweight is the *only reason* for impairment
II) Physical activity	b) The overweight is the *main reason* for impairment
III) Emotional problems	c) The overweight is *a possible reason* for impairment
IV) Pain	d) The overweight is *presumably not the reason* for impairment
V) Social or familial problems	e) The overweight is *not the reason* for impairment
VI) Work capacity	Expectations to changes in aspects of general well-being as a result of weight loss
	a) Much better
	b) Somewhat better
	c) Unchanged
	d) Somewhat worse
	e) Much worse
Follow-up	
Are your expectations to changes in general well-being fulfilled as a result of weight loss	a) Yes
	b) Somewhat
	c) No

#### Anthropometric Measures

Information on weight and height was obtained from the medical record at baseline and follow-up. Height was measured to the nearest 0.5 cm without shoes. Weight was measured to the nearest 100 g with light clothing.

### Statistics

*T*-test and Wilcoxon signed rank test were used to compare continuous variables as appropriate. Multiple linear regression was used to assess the association between fulfilment of expectations and health-related quality of life. P-values below 5 % were considered statistically significant. Analyses were performed using STATA Statistical Software v. 11.2.

## Results

### Study Participants

A total of 238 obese patients had a medical examination at the Department of Medical Endocrinology, which aimed at evaluating the patients’ suitability for bariatric surgery.

Before the first medical examination the patients answered questionnaires assessing health-related quality of life, attributions for impaired general well-being and expectations to changes in general well-being as a result of weight loss.

Patients who had gastric bypass at Aalborg Hospital – Aarhus University Hospital were asked to answer follow-up questions 4 and 12 months after surgery. In total 136 patients were eligible for follow-up 4 months after surgery. Due to incomplete answers and logical errors in SF-36 a total of 31 patients were excluded at 4 months follow-up. At the follow-up point of 12 months 10 patients had incomplete answers in the questionnaires and 8 patients did not turn up for the medical examination and as a consequence no weight information was available. In total 87 patients were included for analysis. Baseline characteristics of participants and non-participants are presented in Table 2. There were no statistical differences between participants and non-participants.

**Table 2:** Baseline characteristics

	Complete follow up after gastric bypass	Lost to follow-up after gastric bypass
*n*	87	49
Age, mean (95 % CI), years	40.9 ± 9.4	39.5 ± 8.8
Gender, male/female	13/74	12/37
Excess weight, mean (95 % CI), kg^a^	58.5 ± 16.7	61.7 ± 18.0
BMI, mean (95 % CI), kg/m^2^	45.6 ± 6.0	46.4 ± 6.5
PCS, mean (95 % CI)	37.5 ± 10.5	36.4 ± 9.5
MCS, mean (95 % CI)	45.1 ± 9.3	44.5 ± 12.0

*PCS* physical component summary at baseline

*MCS* mental component summary at baseline

^a^weight – weight when BMI = 25 kg/m^2^

### Health-Related Quality of Life

At baseline the patients reported low health-related quality of life compared to Danish norm data.

At both follow-up points the physical component summary and the mental component summary were improved as compared to baseline. The health-related quality of life scores are shown in Table 3.

**Table 3:** Health-related quality of life

	Baseline	4 months follow-up	12 months follow-up	National norm
	*n*: 87	*n*: 87	*n*: 87	*n*: 784
	Mean (SD)	Mean (SD)	Mean (SD)	Mean (SD)
PCS	37.51 (10.47)*	49.80 (8.41)** ^#^	51.22 (9.70)^#^	52.63 (7.29)
MCS	45.07 (9.31)*	53.87 (8.72)^#^	51.67 (10.68)^#^	53.55 (8.29)

*PCS* physical component summary

*MCS* mental component summary

*P < 0.0001 compared to national norm data

**P < 0.05 compared to national norm data

^#^ *P* < 0.0001 compared to baseline

### Attributions of Low General Well-being to Obesity

**Fig. 3:**
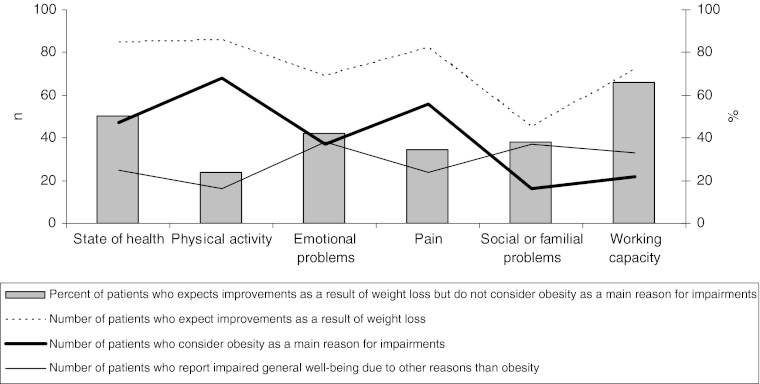
Baseline evaluation of reasons for impairment and expectations as a result of weight loss

At baseline almost all the patients expect improvements in state of health, physical activity and pain as a result of weight loss. This is illustrated by the dotted line in Fig. 3. Furthermore the figure illustrates the number of patients who consider obesity as the main reason for impairment in state of health, physical activity, emotional problems, pain, social or familial problems and working capacity, this is illustrated by the bold line. Finally the numbers of patients who ascribe the limitations to other factors than obesity are illustrated by the solid line. To emphasise the discrepancy between expectations and reality, the percent of patients who expect improvements as a result of weight loss but at the same time do not consider obesity as the reason for their impairments are illustrated by the columns in Fig. 3. Expecting improvements as a result of weight loss, even though the bodyweight is not the main cause of impaired general well-being, was especially present regarding state of health and working capacity.

### Association between Fulfilment of Expectations and Health-related Quality of Life

Compared to the reference group which consists of patients with fulfilled expectations the patients with expectations partly fulfilled had a low mental component summary score −7.3 (−11.3; −3.3). Patients who reported not to have expectations fulfilled also had a low mental component summary score −11.2 (−18.8; −3.5) when compared to their counterparts with fulfilled expectations 4 months after surgery.

At 12 months follow-up patients who reported not to have expectations fulfilled had a low mental component summary score −16.3 (−26.5; −6.2) when compared to their counterparts with fulfilment of expectations. The estimates and corresponding 95 % confidence intervals are shown in Table 4.

**Table 4:** Association between fulfilment of expectations and the mental component summary

	4 months after surgery		12 months after surgery	
	Crude	Adjusted^a^	Crude	Adjusted^a^
	Estimate (95 % CI)	Estimate (95 % CI)	Estimate (95 % CI)	Estimate (95 % CI)
Expectations to change in general well-being fulfilled as a result of weight loss 4 months after gastric bypass				
Yes	Ref	–	Ref	–
Somewhat	−7.3 (−11.2; −3.4)	−7.3 (−11.3; −3.3)	−5.2 (−10.3; −0.04)	−5.0 (−10.7; 0.7)
No	−11.1 (−18.4; −3.8)	−11.2 (−18.8; −3.5)	−15.6 (−24.8; −6.3)	−16.3 (−26.5; −6.2)

^a^Adjusted for age, sex, bmi at follow-up and medical treatment

## Discussion

### Main Findings

The present results of an association between fulfilment of expectations and health-related quality of life at both 4 and 12 months after gastric bypass support Calmans hypothesis of an inverse association between expectations and quality of life (Calman 1984).

### Strengths and Limitations

Our study has methodological strengths and limitations which need to be considered. This is further described below.

### Random Variation

One of the main strengths of our study is the prospective study design of bariatric patients who had a gastric bypass operation between February 2008 and December 2009 at a public university hospital. We used 95 % confidence intervals to indicate the degree of statistical precision for point estimates.

### Selection Problems

At baseline all candidates for gastric bypass answered the questionnaire. Not all patients who were eligible to follow-up did so; consequently the study participants may not represent gastric bypass patients as a whole. Baseline characteristics, represented in Table 2, did not differ between patients with complete data at follow-up and patients who were excluded because of insufficient follow-up data. If having complete data at follow-up was found to be related to a good health-related quality of life the estimates of the outcome, which are represented in Table 3, is said to be overestimated.

As regards the association between fulfilment of expectations and health-related quality of life, incomplete data at follow-up, which may be unequally distributed for the explanatory variable, would not bias the point estimates of the association. The estimates are shown in Table 4.

The essential part when considering relative measures of the association is that the participating patients represent the gastric bypass patients as regards the association, not the distribution of the explanatory variable. We have no reason to believe that the association between fulfilment of expectations and health-related quality of life differs among the included patients and patients without complete data at follow-up.

### Information Problems

Response shift may be an issue in studies with prospective follow-up of the gastric bypass patients’ well-being and health-related quality of life, since the basis on which they evaluate or define these subjective constructs may change over time (Oort et al. 2009; Ring et al. 2005). White et al. described stable goal weights among gastric bypass patients at follow-up points of 6 and 12 months (White et al. 2007). But at present we are not aware of studies describing stable expectations to changes in general well-being.

Selective reporting of problems which the patients believe to be unrelated to obesity may distort the interpretation of the results. However the patients were instructed to include all symptoms and problems irrespective of origin when answering the questionnaires.

### Confounding

We adjusted the analyses for predefined putative confounding factors. However we can not rule out the possibility that unmeasured confounders explain the observed associations.

### Comparison with other Studies

The growing use of quality of life in the medical literature may be ascribed to different factors, for example the ageing of the population whereby chronic diseases become more prevalent combined with the realisation that chronic diseases are seldom cured. These factors emphasise health-related quality of life as an important outcome for patients, clinicians and researchers. Compared to Danish patients who underwent pituitary surgery for clinically nonfunctioning pituitary adenoma (Nielsen et al. 2007) the gastric bypass patients in the present study had low physical and low mental component summary, *P < 0.0001.* Furthermore the gastric bypass patients had a low mental component summary score when compared to Danish patients treated with haemodialysis (Molsted et al. 2004).

In the present study the discrepancy between attributions for low general well-being to obesity and expectations to changes as a result of weight loss is least predominant as regards physical activity and pain. This finding is in accordance with previous published results in a setting of bariatric patients (Pristed et al. 2011). Recently high expectations to weight loss have been reported among gastric bypass patients (Karmali et al. 2011; Wee et al. 2006; White et al. 2007) and in this study we show that gastric bypass patients expect improvements within aspects of general well-being which are impaired due to other reasons than obesity.

In accordance with previous studies we found low health-related quality of life at baseline as compared to national norm data (Choban et al. 1999; Dixon et al. 2001; Larsson et al. 2002; Pristed et al. 2011; Victorzon et al. 2010). Improvements in health-related quality of life were seen at both follow-up points, as seen previously (Choban et al. 1999; Dixon et al. 2001; Karlsson et al. 2007; Pristed et al. 2011).

In agreement with Calman’s conceptual model of quality of life (Calman 1984) we found an association between fulfilment of expectations and the mental component summary and with this finding we found statistical evidence to reject the hypothesis of no association. The conceptual model of quality of life is not empirically tested before in a setting with gastric bypass patients.
